# Draft Genome Sequence of an Endophytic *Micromonospora* sp. Strain, ANENR4, Isolated from the Root of a Peanut Plant (Arachis hypogaea)

**DOI:** 10.1128/mra.00655-22

**Published:** 2022-10-20

**Authors:** Md Majharul Islam, Biraj Sarkar, Pulak Kumar Maiti, Sandip Das, Paulami Dam, Rittick Mondal, Trishanjan Biswas, Abdul Sadat, Arka Pratim Chakraborty, Debnirmalaya Gangopadhyay, Sukhendu Mandal, Ahmet Kati, Amit Kumar Mandal

**Affiliations:** a Laboratory of Molecular Bacteriology, Department of Microbiology, University of Calcutta, Kolkata, West Bengal, India; b Department of Botany, School of Sciences, Durgapur Regional Center, Netaji Subhas Open University, Kolkata, West Bengal, India; c Department of Sericulture, Raiganj University, North Dinajpur, West Bengal, India; d Department of Botany, Raiganj University, North Dinajpur, West Bengal, India; e Experimental Medicine Research and Application Center, University of Health Sciences Turkey, Uskudar, Istanbul, Turkey; f Department of Biotechnology, Institution of Health Sciences, University of Health Sciences Turkey, Uskudar, Istanbul, Turkey; Indiana University, Bloomington

## Abstract

The genus *Micromonospora* was found to occur in a diverse range of habitats. Here, we report the genome sequence of an endophytic strain of *Micromonospora* sp., ANENR4. ANENR4 was isolated from the healthy roots of a peanut (Arachis hypogaea) plant from Egra, West Bengal, India.

## ANNOUNCEMENT

The genus *Micromonospora* is a Gram-positive, sporulating actinobacterium that belongs to the family *Micromonosporaceae* in the order *Micromonosporales* and phylum *Actinobacteria* (1–3). Members of this genus are aerobic, filamentous bacteria that possess unique morphological characteristics, including nonmotile single spores borne directly on the tips of nonfragmented substrate mycelia and the absence of aerial mycelia (4). *Micromonospora* isolates have been obtained from different geographical zones, like soil (5) marine environments (1), sea sands (6), near-shore sediments (7), deep-sea sediments (8), and marine sponges (9), as well as nitrogen-fixing nodules of both leguminous and actinorhizal plants (10). Species of this genus have received a lot of consideration during biosynthetic metabolite screening programs, given its ability to produce various secondary metabolites (11). Plant coinoculation studies revealed that some *Micromonospora* species are able to promote plant growth (12).

The isolated strain ANENR4 was obtained from cultivated peanut crop field in Egra (21°53′58.09′′N, 87°32′16.58′′E), West Bengal, India. For isolation of the endophytes, the healthy root surfaces of peanut were sterilized by washing off the dirt in flowing sterile Milli-Q water, followed by dipping in 70% (v/v) ethanol for 1 min. The roots were then again washed twice in 0.9% (wt/vol) NaCl solution, followed by dipping in 2% (vol/vol) sodium hypochlorite solution (NaClO) for 4 to 5 min, and finally rinsed 5 or 6 times with sterile deionized water. To cross check the complete surface sterilization process, 100 μL of water from the last rinse was added to a starch casein agar plate (1 L of medium contains 10 g starch, 2 g KNO_3_, 2 g NaCl, 2 g K_2_HPO_4_, 0.05 g MgSO_4_·7H_2_O, 0.02 g CaCO_3_, 0.01 g FeSO_4_, 0.3 g casein, and 20 g agar [pH 7.0]) and incubated for 4 to 5 days at 30°C. About 1-cm lengths of the roots were collected and crushed in 10 mL of 0.9% (wt/vol) NaCl solution using sterile mortar and pestle. The extract was then collected and diluted serially. One hundred microliters from each of all the diluted fractions was spread on independent plates containing different solid media, like *Actinomycetes* isolation media (AIA) (1 L of medium contains 5 g glycerol, 4 g sodium propionate, 2 g sodium caseinate, 0.1 g l-asparagine, 0.001 g FeSO_4_·7H_2_O, and 15 g agar [pH 8.1]), starch casein, and yeast extract mannitol agar (YEMA) medium (10 g mannitol, 0.2 g MgSO_4_·7H_2_O, 0.2 g NaCl, 0.5 g K_2_HPO_4_, 1 g yeast extract, and 15 g agar [pH 6.8 ± 0.2]) containing Congo red, and incubated at 30°C. The medium pH was maintained within the range of 7 to 8, and to avoid any fungal growth, cycloheximide (50 μg/mL) was added. The inoculated agar plate was then incubated for 4 to 5 days at 30°C. Thereafter, single colonies were individually picked and transferred to fresh ISP-2 (International *Streptomyces* Project-2) agar plates (10 g malt extract, 4 g yeast extract, 4 g dextrose, and 20 g agar in 1 L of Milli-Q-grade autoclaved water [pH 7.0]) using a sterile inoculating loop to obtain pure colonies through streak plate method.

Strain ANENR4 was grown in ISP-2 broth medium (10 g malt extract, 4 g yeast extract, and 4 g dextrose in 1 L of Milli-Q-grade autoclaved water [pH 7.0]) at 30°C under shaking conditions at 120 rpm for 4 to 5 days. The genomic DNA was isolated from freshly grown cells of ANENR4 as per the standard phenol-chloroform method (13, 14). The paired-end libraries were prepared and sequenced using the Illumina HiSeq X10 platform (AgriGenome Labs Pvt., Ltd., Kochi, Kerala, India), producing a total of 19,907,924 reads with a 2 × 150-bp paired-end read length. The DNA library was prepared using the NEBNext Ultra DNA library prep kit following the manufacturer’s manual. To accomplish data preprocessing, unique reads were first obtained using BBTools v.38.57 (https://sourceforge.net/projects/bbmap) (15). Removal and low-quality end trimming were done using the AdapterRemoval tool v.2.3.1 (https://github.com/MikkelSchubert/adapterremoval) (16). The preprocessed reads were further screened to filter out reads from the probable contaminating plasmid using an in-house database (curated from the public databases, namely, NCBI-NT and NCBI RefSeq, by AgriGenome Labs Pvt., Ltd.), and the unaligned reads were used for assembly preparation. The *de novo* assembly was completed using the Unicycler v.0.4.8 (https://github.com/rrwick/Unicycler) assembler (17). The annotation was carried out employing the NCBI Prokaryotic Genome Annotation Pipeline (PGAP) v.4.13 with the methods “best-placed reference protein set” and “GeneMarkS-21” (18). The assembly produced a draft genome sequence encompassing 66 contigs. The *N*_50_ length is 372,003 bp, and the *L*_50_ count is 07. The estimated genome size is 6,550,221 bp with a 72.9% G+C content and 93.57× coverage. A total of 6,050 coding sequences were annotated, including 5 rRNA genes (5S, 16S, and 23S) and 51 tRNAs. Further focus on the genome of ANENR4 will definitely help in understanding the detailed molecular basis of bioactive secondary metabolite production for future use in medicine and therapeutics.

### Data availability.

This whole-genome shotgun project has been deposited at NCBI under accession number JADKYQ000000000. The version described in this paper is the first version, JADKYQ010000000. The BioSample and BioProject accession numbers are SAMN16604651 and PRJNA673354, respectively. The raw data are available from the Sequence Read Archive (SRA) under accession number SRR18909592.
